# Wound-Healing Markers Revealed by Proximity Extension Assay in Tears of Patients following Glaucoma Surgery

**DOI:** 10.3390/ijms19124096

**Published:** 2018-12-18

**Authors:** Éva Csősz, Noémi Tóth, Eszter Deák, Adrienne Csutak, József Tőzsér

**Affiliations:** 1 Biomarker Research Group, Department of Biochemistry and Molecular Biology, Faculty of Medicine, University of Debrecen, Egyetem ter 1., 4032 Debrecen, Hungary; deak.eszter@med.unideb.hu (E.D.); tozser@med.unideb.hu (J.T.); 2 Proteomics Core Facility, Department of Biochemistry and Molecular Biology, Faculty of Medicine, University of Debrecen, Egyetem ter 1., 4032 Debrecen, Hungary; 3 Department of Ophthalmology, Faculty of Medicine, University of Debrecen, Nagyerdei krt. 98., 4032 Debrecen, Hungary; tothnoemi111@gmail.com (N.T.); acsutak@med.unideb.hu (A.C.)

**Keywords:** proximity extension assay, tear, glaucoma, wound healing, inflammation

## Abstract

Tears are a constantly available and highly valuable body fluid collectable by non-invasive techniques. Although it can give information on ocular status and be used for follow-ups, tear analysis is challenging due to the low amount of sample that is available. Proximity extension assay (PEA) allows for a sensitive and scalable analysis of multiple proteins in a single run from a one-µL sample, so we applied this technique and examined the amount of 184 proteins in tears collected at different time points after trabeculectomy. The success rate of this surgical intervention highly depends on proper wound healing; therefore, information on the process is indispensable. We observed significantly higher levels of IL-6 and MMP1 at the early time points (day one, two, and four) following trabeculectomy, and the protein amounts went back to the level observed before the surgery three months after the intervention. Patients with or without complications were tested, and proteins that have roles in the immune response and wound healing could be observed with altered frequency and amounts in the cases of patients with complications. Our results highlight the importance of inflammation in wound-healing complications, and at the same time, indicate the utility of PEA in tear analysis.

## 1. Introduction

Glaucoma is a multifactorial neurodegenerative eye disease that affects millions of people and is a leading cause of blindness worldwide [1]. In glaucoma, the neuropathy of the optic nerve and the progressive and irreversible loss of retinal ganglion cells can be observed, resulting in the atrophy of the optic nerve and the loss of visual functions, leading to blindness [2,3]. The most important form of treatment is the reduction of the increased intraocular pressure (IOP), which can delay the glaucoma progression and prevent visual field loss. Most of the patients are initially treated and well controlled with topical medication (eye drops), but surgery becomes necessary when the desired IOP cannot be reached. Some minimal invasive microsurgery procedures are available, but the most widely used gold standard surgical intervention in open angle glaucoma is still filtration surgery. The latter involves trabeculectomy, which is an invasive procedure associated with a relatively high complication and failure rate [4,5]. During trabeculectomy, a part of the trabecular meshwork is removed, and a channel is created between the anterior chamber and the subconjunctival space, leading to controlled leaking of the aqueous humor, and thus the lowering of the IOP. One of the key features of the success of trabeculectomy is the wound healing, which might be impaired, making the postoperative IOP control impossible [4].

Wound healing is a well-organized cascade of closely linked processes starting with a coagulative and inflammatory phase, followed by the proliferative and repair phase, and ending with the remodeling phase [6]. Ocular wound healing differs from that observed in case of skin [7]; right after the injury in the first few hours in the epithelial cell layer, the phases of latency, migration, and proliferation occur with the proliferation phase peaking around 24 hours after the injury [8,9]. The ATP released from the injured cells and the damage-associated molecular patterns made available by the injury increase the apoptosis and toll-like receptor signaling, which leads to the production of pro-inflammatory cytokines such as interleukins IL-1α and β, IL-6, and tumor necrosis factor α (TNFα) [10,11]. The release of matrix metalloproteinases (MMPs) triggers the extracellular matrix rearrangements, making possible fibronectin polymerization, the remodeling of cellular junctions, the formation of focal contacts at wound margins, and the migration of cells to cover the wound [8,12]. These steps are followed by cell proliferation, the formation of a basement membrane, and the restoration of barrier functions orchestrated by keratinocyte growth factor (KGF), hepatocyte growth factor (HGF), epidermal growth factor (EGF), platelet-derived growth factors (PDGF), neural growth factor (NGF), transforming growth factors (TGF), and cytokines (mainly IL-1, IL-6, and IL-8, as reviewed by [8,11,13]). At the same time, keratocyte apoptosis in the stroma is followed by the recruitment of immune cells and keratocyte transformation to fibroblasts and myofibroblasts [14,15,16]. The transformed and the newly formed cells migrate to the site of injury to fill up and close the wound. Less information is available regarding the endothelial cells in which the migration of the endothelial cells to fill up the gap, and the secretion of basement membrane was observed to happen in an approximately six hours to time interval of several days [8,12].

The phases of ocular wound healing are regulated by the tightly controlled appearance of cytokines and chemokines released during injury. These molecules in turn lead to the appearance of other cytokines and growth factors, making possible the coordination of molecular events that is characteristic to the latency, migration, and proliferation phases both in the epithelial and stromal layers. Epithelial cells express interleukins (IL), tumor necrosis factor (TNF) α, and growth factors under normal conditions, and they are activated/released during the injury [11]. Growth factors and cytokines from tears that are released upon the injury, which in turn increase the production of other cytokines and growth factors [9,17]. EGF and PDGF are released during the injury, and the amounts of KGF and HGF also are increased [13,17]. Meanwhile, the controlled activation of MMPs makes the large extracellular matrix rearrangements that are required for cell migration and wound closure possible. The wound-healing process ends with a slow and long remodeling phase that lasts for up to a year and ends up, under normal conditions, with the scarless healing that is necessary for proper vision [8,12,18].

Each step of the process can be affected, leading to complications and ineffective surgical intervention. Reepithelization typically occurs mainly three to five days after surgery [12], but complications appear later (weeks or months) after surgery [19,20], highlighting the importance of the early events of wound healing.

In order to get more information on the ocular wound-healing process following trabeculectomy, we decided to use tears, the accessible body fluid that can be collected in tiny amounts by non-invasive techniques. To examine multiple proteins in the same sample, methods permitting multiplexing are required. Such methods can be based on mass spectrometry, immunological detection, or a combination of different methods. Immunological detection in most of the settings is more sensitive compared to mass spectrometry, but the dynamic range of the mass spectrometry usually overcomes that which is achievable by the immunological methods [21], and moreover, it is the only unbiased method. At the same time, it is difficult to work reliably in many cases with many different biological materials by mass spectrometry. A novel approach with exceptional specificity and scalability is the combination of antibody-based detection with the well-defined methods that are used during quantitative polymerase chain reaction (qPCR). The proximity extension assay (PEA) uses two polyclonal antibodies raised against the protein of interest and linked to complementary oligonucleotides. When the two antibodies bind the protein of interest, oligonucleotides hybridize. After hybridization, DNA polymerase is added, and qPCR is carried out, permitting the quantification of the protein-specific oligonucleotide sequences. The results are given as normalized expression units (NPX) and can provide the relative quantification of proteins [22]. This method, by combining two, individually powerful analysis techniques, makes relative quantification of multiple proteins possible in very low sample amounts, providing an effective tool for the analysis of body fluids available in low volumes [23,24,25,26].

During this work, our aim was to apply the PEA method on tear analysis to get more information on the amounts of proteins that have roles in the different phases of the ocular wound-healing process, with highlights on complications following trabeculectomy.

## 2. Results and Discussion

The tightly regulated process of ocular wound healing makes the scarless healing of tissue injuries possible, and involves a multitude of molecules playing role in the regulation of the biochemical events of the wound-healing steps. In order to examine the relative amount of multiple tear molecules, giving information on a novel method for ocular wound healing, PEA was applied for the analysis of tear proteins. This method permits the analysis of up to 92 proteins per panel using only one µL of body fluid, which can be extremely beneficial in the case of body fluids that are collectable in low volume such as tears, where the typical amount collected is 5–10 µL [27].

### 2.1. Tear Sample Analysis by PEA

**Figure 1:**
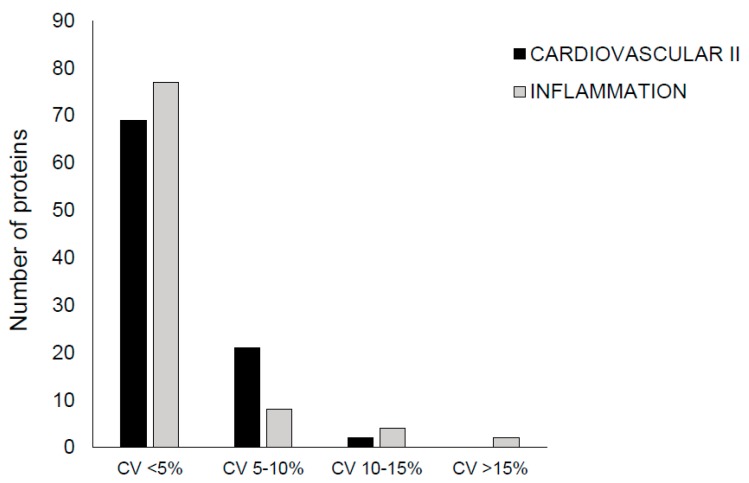
Intraassay %CV distribution. The number of proteins with %CV within defined intervals is indicated in case of Cardiovascular II and Inflammation panels.

Tear samples were analyzed by PEA at Olink Proteomics (Uppsala, Sweden) using the cardiovascular (CVD) II and the inflammation panels. Each panel included 92 proteins and four internal controls, providing information about 184 proteins altogether (Table S1). Due to technical issues identified with the brain-derived neurotrophic factor (BDNF) assay, the BDNF protein could not be analyzed. According to our data, 11 out of the analyzed 184 proteins do not appear in tears. These proteins are fibroblast growth factor 5, interferon gamma, interleukins IL2, IL20, and IL24, interleukin receptors IL2RB, IL15RA, and IL22RA1, signaling lymphocytic activation molecule (SLAM) family member 1, thymic stromal lymphopoietin (TSLP), and tumor necrosis factor. Internal controls were added to each sample to monitor the quality of assay performance and the quality of individual samples. Quality control (QC) was performed in two steps: i) each sample plate was evaluated based on the standard deviation (SD) of the internal controls, and only data from the sample plate that pass quality control (SD < 0.2 NPX) were reported, and ii) the quality of each sample was assessed by evaluating the deviation from the median value of the controls for each individual sample. Samples that deviate less than 0.3 NPX from the median pass the quality control. In the CVD II and inflammation panels, 55 out of 60 and 57 out of 60 samples passed the QC, respectively (Table S2). Most proteins of the could be measured with <5% coefficient of variation (CV) with a typical intraassay CV of 4% (Figure 1).

In the case of the CVD II panel, 60% of the proteins (55 out of 92) could be detected in more than 75% of the samples, while in case of the inflammation panel, this value was 45% (41 proteins out of 92) (Table S2). Compared to the more than 90% detectability for proteins included in the CVD II panel and >75% for proteins included in the inflammation panel observed in plasma, the values obtained for tear analysis are lower, but it should be mentioned that the protein composition of tears was substantially different from that of plasma [28]. At the same time, NPX values may differ depending on the sample preparation methods, sample matrices, or the pathological conditions that are present. However, based on our data, both the CVD II and inflammation panels that are used primarily for plasma protein analysis work well in case of tears, and they provide easy-to-use tools for the multiplex analysis of proteins from small amounts of samples. This is of extremely high importance, because the amount of basal tear that is available is usually low, in some cases hindering the administration of replicates in very sensitive mass spectrometry analyses. Reflex tear can be collected in higher amounts, but its quality is significantly different compared to basal tear [29]. Considering the qualitative and quantitative differences between the basal and reflex tear, arguments against pooling for tear analysis, and that the method does not require technical replicates due to the application of proper controls, PEA can be considered a method of choice for the analysis of individual, basal tear samples.

### 2.2. Examination of Protein-Level Changes after Trabeculectomy

To interrogate the role that selected tear proteins have in wound healing, the amount of 184 proteins was analyzed in a follow-up study in tear samples originating from patients who underwent trabeculectomy. The starting point of the study was day zero, when the samples were collected before the surgical intervention. By examining changes in the level of interleukins, growth factors, extracellular matrix components, and other proteins with possible roles in wound healing in early and later time points (postoperative day one, day two, day four, and three months), our aim was to understand the subtle events related to the different phases of wound healing. After reviewing patient records, we could not find bleb leak, hypotony, hyphema, choroidal effusion, choroidal hemorrhage, blebitis, or endophthalmitis. In the case of three patients, the surgical intervention could not lower the high IOP properly, and the administration of IOP-lowering eye drops was required as indicated in Table S3. This latter group was designated as the “group with complications”.

The amount and the frequency of the 184 tested proteins were examined in the groups with and without complications using a step-by-step procedure. First, a qualitative analysis was carried out, followed by quantitative analysis, heat map analysis, hierarchical clustering, and statistical analysis. The proteins with altered frequency or the amount between the groups with and without complications were subjected to functional analysis. First, the network of proteins was created with the help of String (https://string-db.org/) and analyzed, followed by Gene Ontology (GO) enrichment analysis. The String database provides an easy-to-use tool for analyzing protein–protein interaction networks, and gives information on the structure and function of proteins as retrieved from curated databases such as UniProt, PDB, Ensembl, etc. The analysis of the enriched GO functions can also be done; the enriched functions, the false discovery rate, and the number of proteins participating in the specified functions can be listed [30,31].

**Figure 2:**
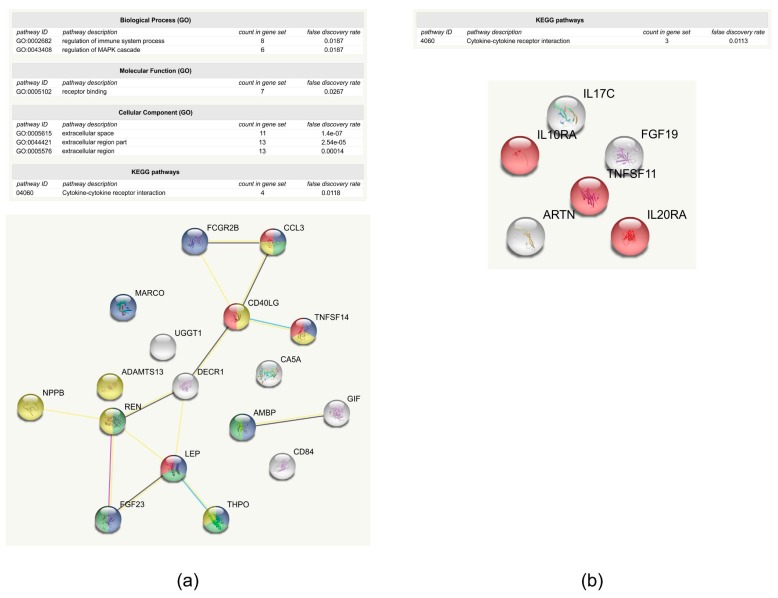
Proteins present with different frequency in the groups. (**a**) Proteins that are less likely to be present in samples belonging to a group with complications; (**b**) proteins that are more likely to be present in samples belonging to a group with complications. In both cases, the upper part of the figure shows the enrichment table generated by String. In the enrichment table, the name and GO code of the enriched pathway, the number of proteins belonging to the pathway, and the false discovery rate are indicated. The lower part of the figures show the network generated by String, with each ball representing a protein, and lines representing protein–protein interactions. Proteins colored with red participate in cytokine–cytokine receptor interaction, the blue color represents the proteins that are responsible for the regulation of immune system processes, and proteins colored with green participate in the regulation of the MAPK cascade, while the ones colored with yellow are responsible for receptor binding. Proteins are represented by their gene name.

Proteins where there was at least a 20% difference in their frequency between the groups with or without complication (Table 1, Table S4) were introduced into String network analysis (Figure 2). The network of proteins that appear less likely in the samples of patients belonging to group with complications contains 17 proteins. The A disintegrin and metallopeptidase with thrombospondin type 1 motif 13 (ADAMTS13), alpha-1-microglobulin/bikunin precursor (AMBP), chemokine (C-C motif) ligand 3 (CCL3), CD40 ligand, CD84 molecule, carbonic anhydrase (CA5A), 2,4-dienoyl CoA reductase 1 (DECR1), IgG Fc fragment low affinity receptor (FCGR2B), fibroblast growth factor 23 (FGF23), gastric intrinsic factor (GIF), leptin (LEP), macrophage receptor with collagenous structure (MARCO), atrial natriuretic factor (BNP/NPPB), renin (REN), thrombopoietin (THPO), tumor necrosis factor superfamily member 14 (TNFSF14), and uridine diphosphate-glucose glycoprotein glucosyltransferase 1 (UGGT1) appear less likely in the samples originating from patients with complications. The enriched GO functions are related to receptor binding, the regulation of MAPK cascade, and immune regulation (Figure 2a). CD84 has a role in modulating the activation and differentiation of immune cells, the mitochondrial DECR1 and CA5A participate in metabolism, and the gastric intrinsic factor has a role in cyanocobalamin absorption. According to functional analysis, UGGT1 has a role in the quality control of protein folding. It recognizes glycoproteins with minor folding defects, reglucosylates them, and facilitates their recycling to the endoplasmic reticulum [32]. Eleven of these proteins were already found to be related to wound healing (Table 1). It was shown that leptin and NPPB promote wound healing in the skin [33,34,35], and FGFs facilitate cell migration and regeneration during wound healing [36]. In case of renin, data are not clear; the renin-angiotensin system is implicated in wound healing but, depending on the conditions, it drives the system either to pro-inflammatory, pro-proliferative, and pro-fibrotic, or anti-inflammatory, anti-proliferative, and anti-fibrotic directions [37]. In the case of the CD40 ligand, TNFSF14, CCL3, and ADAMTS13, it was shown that their lack or decreased level leads to impaired wound healing. The lack of CD40L is related to increased collagen deposition, while the lack of ADAMTS13 increases the extravasation of neutrophils in the early phase of wound healing [38,39]. Platelet hyperactivity, impaired wound healing, and the accumulation of immune cells along with high cytokine levels were observed upon the lack of TNFSF14 [40,41], and reduced levels of CCL3 were observed in impaired wound healing [42]. THPO has a role in platelet synthesis, but it was observed that treatment with the THPO receptor agonist slightly delayed the healing rate in wounded mice [43]. Bikunin or inter-alpha trypsin inhibitor is related to impaired wound healing; more pathological-appearing cells were observed after naphthalene injury in inter-alpha trypsin inhibitor-deficient mice significantly [44]. There are no data regarding the CA5A, but it was shown that the carbonic anhydrase is expressed in skin during wound healing, and its reduced levels were observed in keloid scars [45]. At this point, we cannot properly address why the reduced occurrence of leptin, NPPB, and FGF23 was observed in the samples originating from the group with complications, but the reduced frequency of the other eight proteins already related to wound healing can be explained; their reduced level was linked to wound-healing problems, as it is the case in the group with complications. It also should be noted that data regarding the link between the examined proteins and wound healing are mainly from studies on skin or airways, and conditions in the eye may differ from those observed in other parts of the body. IL17C, IL10RA, IL20RA, FGF19, artemin (ARTN), and TNFSF11 were found to appear more frequently in the samples of patients with complications (Table 1). String analysis shows that these proteins do not interact with each other, and the only enriched pathway was cytokine-binding (Figure 2b). It was already observed by different groups that IL17C plays an important role in the innate immunity of the epithelium by stimulating the production of antibacterial and immunomodulatory peptides through the NF-kappa-B and MAPK pathways [46], and the overexpression of IL17C is associated with psoriasiform skin [47]. IL20 and its receptor IL20RA promote the epithelial wound healing in mouse cornea [48], IL10RA promotes the survival of myeloid cells [49], and TNFSF11, as a ligand for NF-kappa-B, promotes cell proliferation [50]. Artemin was shown to enhance the migrating capability in wound healing, and many members of the FGF family are applied as drugs helping the wound healing [51,52]. The increased frequency of proteins having roles in cell migration, cell proliferation, and enhancing wound healing in patients with complications might give an explanation of the presence of complication being possibly related to the early closure of the channel generated during trabeculectomy.

**Figure 3:**
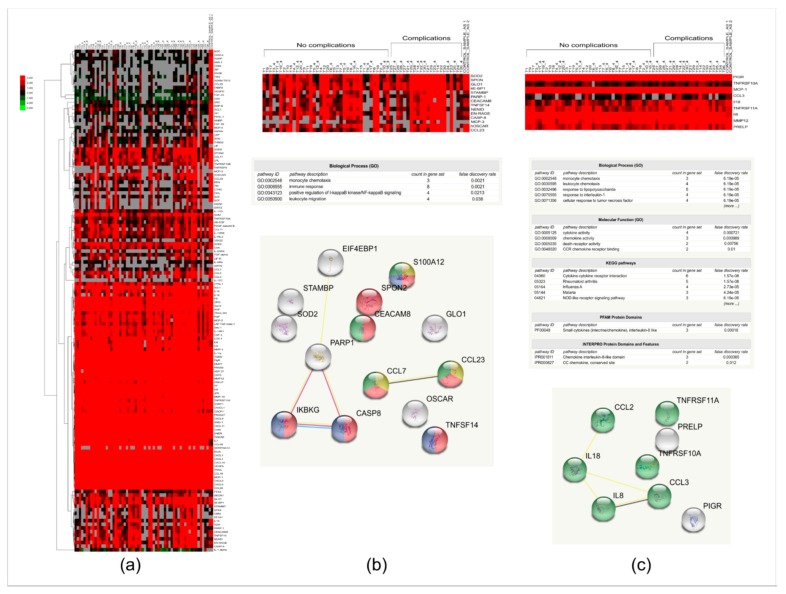
Cluster analysis and heat map of proteins analyzed in tears of patients with glaucoma who underwent trabeculectomy. (**a**) Relative protein amount (NPX) in case of proteins with higher than 30% detectability characteristic for each sample; (**b**) a magnified image of the heat map for proteins showing a higher expression level in the samples belonging to the group with complications is shown, along with the network and the enriched GO terms and pathways. In the enrichment table, the name and GO code of the enriched pathway, the number of proteins belonging to the pathway, and the false discovery rate are indicated. In the network, each ball represents a protein. Proteins colored with yellow participate in monocyte chemotaxis, while those colored with red have a role in the immune response, proteins with the blue color participate in the regulation of NF-kappa B signaling, and those colored with green have a role in leukocyte migration; (**c**) a magnified image of the heat map for proteins showing significantly altered expression levels in samples belonging to group with complications is shown, along with the network and the enriched GO terms and pathways. In the enrichment table, the name and GO code of the enriched pathway, the number of proteins belonging to the pathway, and the false discovery rate are indicated. Proteins colored with green participate in cytokine–cytokine receptor interaction.

In order to have a more in-depth analysis, the qualitative examination was followed by quantitative analysis. The 184 proteins were examined, and 46 of the proteins could be detected in less than 30% of the samples (Table S2), so these proteins were excluded from further statistical analyses. The changes in the relative amount of the proteins were visualized on a heat map (Figure 3a). Hierarchical clustering of the data revealed that, based on these protein signatures, the group with complications cannot be unanimously differentiated from the group without complications. However, higher protein amounts could be observed in case of 14 proteins in the samples belonging to the group with complications (Figure 3b). Functional analysis of these proteins shows an enrichment of proteins belonging to immune response GO (GO:0006955) function. Caspase 8 has a role in apoptosis, protein S100A12, TNF superfamily member 14, monocyte chemotactic protein 3 (MCP-3/CCL7), chemokine (C-C motif) ligand 23 (CCL23), and NF-kappa-B essential modulator (NEMO/IKKBG) have pro-inflammatory functions, while the carcinoembryonic antigen-related cell adhesion molecule 8 (CEACAM8) and spondin 2 have a role in cell adhesion. The osteoclast-associated immunoglobulin-like receptor (OSCAR) participates in osteoclast differentiation, but also functions in neutrophil degranulation, and can activate TNF-α release from inflammatory monocytes [53]. Superoxide dismutase (SOD2) and glyoxalase 1 (GLO1) may have protective roles preventing the accumulation of free radicals and advanced glycation end products (AGEs), and their reduced activity or expression was found to be associated with delayed wound healing [54,55,56,57]. In a corneal wound healing model, the expression of poly-ADP ribose polymerase-1 (PARP-1) was shown to be upregulated by fibronectin, while PARP-1 activation in skin was associated with delayed wound healing [58,59]. The increased amount of PARP-1, osteoclast-associated immunoglobulin-like receptor, SOD2, and GLO1 may indicate the presence of additional stress factors in the group where complications are present. The signal transducing adapter molecule (STAM)-binding protein initiates cell growth and cell proliferation, while the eukaryotic initiation factor EIF4EBP1 has a role in the regulation of translation; both are needed for the increased cell proliferation that is required for wound closure [60]. The increased amounts of molecules whose decrease was associated with the delayed wound healing (SOD2, GLO1, PARP-1) and of those molecules that have a role in proliferation (STAM-binding protein, EIF4EBP1) might reflect premature wound healing, leading to the ineffective trabeculectomy that is characteristic in the group with complications.

Proteins are represented by their abbreviations as follows: 4E-BP1 = EIF4EBP1: eukaryotic translation initiation factor 4E binding protein 1, CASP8: caspase 8, CCL3: chemokine (C-C motif) ligand 3, CCL23: chemokine (C-C motif) ligand 23, CEACAM8: carcinoembryonic antigen-related cell adhesion molecule 8, EN-RAGE = S100A12: S100 calcium binding protein A12, IL8: interleukin 8, IL18: interleukin 18, GLO1: glyoxalase 1, MCP-1 = CCL2: chemokine (C-C motif) ligand 2, MCP-3 = CCL7: chemokine (C-C motif) ligand 7, MMP12: macrophage metalloelastase, NEMO = IKBKG- NF-kappa-B essential modulator, OSCAR: osteoclast associated, immunoglobulin-like receptor, PARP1: poly (ADP-ribose) polymerase 1, PIGR: polymeric immunoglobulin receptor, PRELP: proline/arginine-rich end leucine-rich repeat protein, SOD2: superoxide dismutase 2, SPON2: spondin 2, STAMBP: STAM binding protein, TNFSF14: tumor necrosis factor (ligand) superfamily, member 14, TNFRSF10A: tumor necrosis factor receptor superfamily, member 10a, TNSFRSF11A: tumor necrosis factor receptor superfamily, member 11a. Note that the presence of different abbreviations for the same protein on panel b and c (4E-BP1—EIF4EBP1, EN-RAGE—S100A12, NEMO—IKBKG, MCP-3—CCL7, MCP-1—CCL2) are due to the utilization of different protein databases by the different software tools.

According to a non-parametric Mann–Whitney U-test, nine proteins showed statistically significant differences between the two groups (Table S4). All of the proteins except for the polymeric immunoglobulin receptor (PIGR) showed an increase in the amount in samples belonging to the group with complications. The network analysis revealed a network with few connections, and the enriched pathways were related to immune response (Figure 3c). MMP12 could not be visualized in the network, because the String database did not contain this protein at the time of analysis. IL8, IL18, MCP-1, CCL3, and the TNF receptor superfamily members 10A and 11A participate in cytokine–cytokine receptor interaction, while PIGR has a role in IgA and IgM binding, and the proline/arginine-rich and leucine-rich repeat protein has a role in extracellular matrix assembly [61]. MMP12 was found to be responsible for ocular wound healing, promoting the early repair process of the corneal epithelium by enhancing epithelial cell migration and neutrophil infiltration [62]. As it was expected, the results of both the qualitative and quantitative analyses show that proteins that have a role in the immune response and wound healing were changed in case of patients with complications. In order to get more information on the events regulating the wound healing and be able to monitor the effect of the time and/or complication on the relative protein levels, a rigorous in-depth statistical analysis using a linear mixed model was performed.

**Figure 4:**
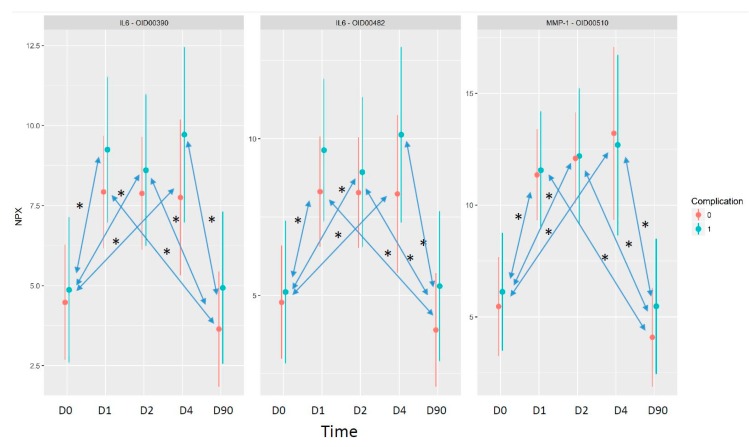
NPX values over the time in the case of proteins with statistically significant differences. The “*x*” axis shows the time points, and the “*y*” axis shows the mean NPX values in the cases of patients with complications (blue color) and without complications (red color) following trabeculectomy. All of the time points were compared to each other; arrows and * indicates statistical significance *p* < 0.05.

Regarding the effect of time, the level of IL6 and MMP-1 changed in a statistically significant way, according to our data. The level of both IL6 and MMP-1 increased in a statistically significant manner on postoperative day one, remained high on days two and four, and went back to the original level three months after the surgical intervention (Figure 4). The changes between day zero and day one, day two, and day four, respectively, were statistically significant, as were the changes between day one, day two, and day four, respectively, and at three months (day 90) as well. The changes between the group with complications and group without complications were not statistically significant.

**Figure 5:**
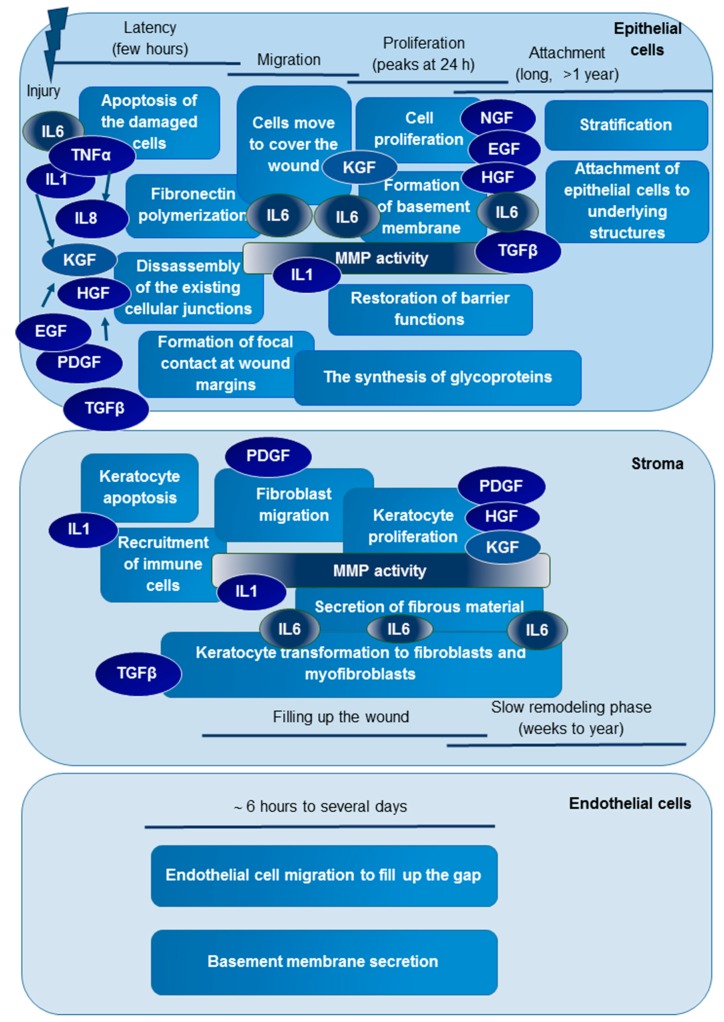
Phases of ocular wound healing. The events that are characteristic for the different phases of ocular wound healing and the regulator molecules are marked with rectangles with ovals, respectively. The dark blue cytokines and growth factors were examined in this study, and those with the black color were found to show statistically significant changes.

IL6 was present in both panels; in this way, it was measured twice, and the very similar behavior of this protein in the two assays provides further evidence for the good replicability of the assays. IL6 has a role in the inflammatory part of wound healing, and is released right after ocular injury [8]. It is known that upregulating the integrin-type fibronectin receptor IL-6 facilitates epithelial cell migration, stimulates the attachment of corneal epithelial cells to collagen type IV and laminin matrices, and may influence the production of fibrotic material by activated keratocytes [63]. The increased level of IL6 observed in tears at postoperative days one, two, and four most probably reflects the higher levels of this cytokine, which is characteristic for the migration and proliferation phases of ocular wound healing (Figure 5). At the same time, the higher level of IL6 that was observed in the tears of patients with complications at all of the postoperative time points supports our previous observation that an increase in the relative amount of proteins participating in immune reaction is detectable in samples originating from patients with complications.

Following injury in the first few hours in the lag or latency phase, the released cytokines (mainly IL-1, IL-6, TNF-α, and IL-8) orchestrate the early events of epithelial wound healing. The damaged cells are dying mainly by apoptosis, and the recruited immune cells help the debridement and the clearance of apoptotic cells. MMPs are activated by IL-1 and other factors, and an extensive extracellular matrix rearrangement starts. The epidermal (EGF), hepatocyte (HGF), keratocyte (KGF), platelet-derived (PDGF), and nerve (NGF) growth factors that are released upon injury or by the action of cytokines help the wound-healing process. During this phase, some of the existing cellular junctions are removed; there is a fibronectin polymerization to help the cell migration, and focal contacts are formed at the wound margin, preparing the conditions for cell migration. In the migration phase, cells migrate to the site of the wound to cover the wound bed. The migration starts approximately five hours after the injury, and is directed by IL-6, KGF, HGF, and PDGF, which is followed by cell proliferation stimulated by the strong mitogenic effect of growth factors. At the same time, extensive synthetic processes are taking place, and the formation of the basement membrane and restoration of the barrier functions happen.

In the stroma, injury is followed by the keratocyte apoptosis and recruitment of the immune cells. IL-1 and TGFβ released in the epithelial cell layer diffuse to the stroma due to the defects of the basement membrane, and regulate the early events of stromal wound healing. The immune cells and keratocytes transform to fibroblasts and myofibroblasts mainly upon the action of TGFβ, and migrate to the site of the injury to fill up the wound. Meanwhile, the keratocytes and fibroblasts secrete growth factors that help the cell proliferation both in the stroma and the epithelial cell layer. The stromal and the epithelial wound healing ends with a slow and long remodeling phase. During this phase in the epithelial cell layer, the stratification of the cells happens, and the firm adherence of the cells to the underlying structures is reestablished. In the stroma, there is an extensive collagen remodeling, and the myofibroblasts disappear. Regarding the endothelial injury, cell migration is the most important process; the endothelial cells migrate to the site of injury, fill up the gap, and secrete a new basement membrane to restore the barrier functions, if necessary.

MMPs have a role in extensive extracellular matrix (ECM) remodeling, which is required for proper wound healing. They have a prominent role in the migration phase, helping cell migration, the recruitment of immune cells, and the production of new fibrotic material (Figure 5). We could observe the increase of MMP-1 in the early postoperative time points, when the matrix metalloproteinase activity promotes the rearrangement of the extracellular matrix, the disintegration of the cell–ECM junctions, the initiation of cell migration, and a decline in the MMP-1 amount at a later time point.

According to our data, both IL6 and MMP-1 have a role not only in the latency and migration phases of ocular wound healing, but also in the proliferation phase and possibly at the early events of the attachment and slow remodeling phases (Figure 5).

**Figure 6:**
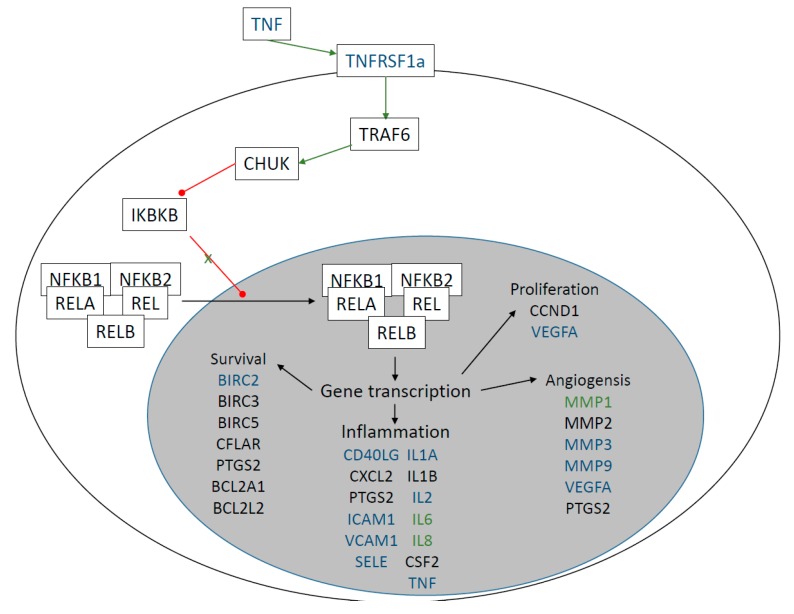
Photodynamic therapy-induced pathway. The proteins highlighted with blue are the proteins that are present in the analyzed panels, while the proteins marked with green indicate those that show statistically significant changes. Green arrows indicate activation, while red arrows indicate inhibition. The pathway was drawn based on Wikipathway WP3617 (www.wikipathways.org/index.php/Pathway:WP3617).

Pathway analysis showed that the relevant pathway involving both IL6 and MMP-1 was the “photodynamic therapy-induced” pathway (Figure 6). According to this pathway, NF-kappa B signaling is activated, which in turn activates among the others the expression of interleukins such as IL6 and of matrix metalloproteinases such as MMP-1. When this pathway is activated in tumor cells, it leads to an inflammation followed by leukocyte migration into the tumor tissue, leading to the apoptosis of the tumor cells [64]. During ocular wound healing, the damaged cells die by apoptosis and necrosis, and the immune cells are recruited to help the tissue regeneration and the clearance of dead cells [8]. The higher activity of this pathway in the first few days (days one, two, and four) after surgery might be responsible for the inflammatory part of the wound-healing process.

Data of the qualitative and quantitative analyses are in line with each other and in accordance with the literature data that indicate a different wound-healing balance in the two groups. In the group with complications, the lower frequency of proteins having a role in the regulation of wound healing and of CA, whose low levels were found in keloid scar formation, was observed. At the same time, higher levels of proteins having a role in immune response, cell proliferation, ECM rearrangements, wound healing, and proteins whose low levels were associated with delayed wound healing (ex. SOD2, GLO1), indicate a more pronounced wound healing compared to the group without complications. Although our data show that the presence and the amount of proteins that have roles in the immune reaction and wound healing are altered in the case of patients with complications, these data have to be considered carefully, since the number of patients recruited into the study was relatively low. New studies with larger number of patients are needed to confirm the increased level of these proteins as possibly responsible for the appearance of complications. Considering that the inflammation is relatively manageable from a therapeutic point of view, these data, if validated on a larger sample size, might help improve the later outcomes of trabeculectomy.

## 3. Materials and Methods

### 3.1. Subjects and Tear Sample Collection

The sample collection was done in accordance with the Declaration of Helsinki, and was approved by the Ethical Committee of the University of Debrecen (approval number: 4234-2014, 11/11/2014). Recruited subjects (six females, two males) were patients of the Department of Ophthalmology, Faculty of Medicine, University of Debrecen, and gave written informed consent for sample collection. All of the patients underwent trabeculectomy surgery to reduce intraocular pressure at the Department of Ophthalmology, Faculty of Medicine, University of Debrecen. A follow-up study including eight patients was carried out, and tear samples were collected before trabeculectomy (day 0), at day one, day four, and three months after the surgery. One of the patients did not present on the three-month control; as a result, the sample could be collected only at the 10-month time point after the surgery. Exclusion criteria were the presence of autoimmune disease and/or any ocular surface disease other than glaucoma. The non-invasive tear collection was carried out before the trabeculectomy using sterile glass capillary tubes (VWR Ltd., Radnor, PA, USA) for two minutes from the lateral inferior meniscus without local anesthesia or stimulation [65]. Tear samples were centrifuged at 4 °C at 2.4× *g* for 10 min in a benchtop Eppendorf centrifuge; then, the supernatants were aliquoted to five-µL aliquots and deep frozen and stored at −70 °C until analysis. Protein concentration of tear samples was determined using the Bradford method [66]. At the time of tear collection, an ophthalmological examination was also carried out.

### 3.2. Relative Quantification of 186 Proteins in Tear by Proximity Extension Assay

Proteins of interest were selected based on previous experiments, and the scientific literature was analyzed by the analysis service of Olink Proteomics (Uppsala, Sweden). Four µL of tears in the case of each sample was shipped on dry ice to Olink Proteomics, and one µL of undiluted and 4x diluted tears, respectively, was used for the analyses. The relative protein quantification was performed using PEA-based panels. The proteins of interest could be found in the inflammation panel and CVD panel II, so these two panels were analyzed. Each panel was able to simultaneously analyze 92 selected proteins, as listed in Supplementary Table S1. Using proper internal and external controls, the raw values were normalized for variation between and within runs, and were converted into normalized protein expression units (NPX), which are arbitrary units on log2 scale, allowing for the relative quantification of proteins this way.

### 3.3. Functional Analysis

The hierarchical clustering of proteins detected in more than 30% of the samples was performed using Gene Cluster 3.0 (http://cluster2.software.informer.com/), and the heat map was created with Java TreeView version 1.1.6r4 [67]. Before clustering, no filtering or adjustment of the data was performed, the distance/similarity measures were based on the Pearson correlation, and the clustering was done by complete linkage analysis. The network of selected proteins was drawn with String 10.5 [30,31] using default settings and medium stringency, and the proteins belonging to the enriched GO functions were highlighted.

Pathway analysis was performed on proteins with statistically significant changes using the Wikipathways (https://www.wikipathways.org/index.php/WikiPathways) search function. The pathways were manually evaluated for their relevance.

### 3.4. Statistical Analysis

A non-parametric Mann–Whitney U-test was applied to compare the mean NPX values that were characteristic for the two groups. The statistical analysis was performed using SPSS 25.0 (IBM Inc., Armonk, NY, USA). For the examination of the effect of surgery and/or time on the relative protein amounts, a linear mixed model and ANOVA was applied using ImerTest function in R [68]. Sixty samples from eight different patients at four different time points were included in the analyses. Samples that did not pass quality control (QC) as defined by Olink were included in the analysis, since their distribution did not appear to differ from the other samples. Proteins with lower than 30% detectability were removed from the analysis, resulting in 138 out of 184 proteins.

Time point and complications were included as fixed effects, and were considered variables of interest. The subject ID was included as a random effect, estimating and controlling for the base protein expression level in each subject. Due to the low number of patients recruited, the effect of gender was not examined. *p*-Values for the fixed effect were estimated using Satterthwaite’s approximation of degrees of freedom, and were corrected for multiple testing using the Benjamini–Hochberg approach [69]. For each significant assay, a post hoc test was performed to estimate the population mean and pairwise differences between groups. Post hoc p-values were calculated using the Tukey method [70].

## 4. Conclusions

We could observe that proteins having a role in immune response and wound healing appear with altered frequency and/or amounts in the samples originating from patients with complications following glaucoma surgery compared to samples from patients with no complications. Further verification and the extension of the study with a higher number of recruited patients are required in order to validate our data and possibly identify new diagnostic and therapeutic target proteins. Based on our data, PEA is a method that is suitable for the analysis of tear samples, and can be a method of choice in the examination of basal tear permitting the relative quantification of hundreds of proteins in individual tear samples.

## Figures and Tables

**Table 1:** List of proteins with altered abundance in the group with complications. The percent of samples expressing the indicated protein in the group with and without complications is listed in the case of each protein where the difference was more than 20% between the two groups. The direction of change in the group with complication is indicated along with biological function and the role in wound healing in the case of each protein. * indicates the enriched Gene Ontology (GO) function calculated by String, and # indicates function as listed in String. ADAMST13: A disintegrin and metallopeptidase with thrombospondin type 1 motif 13, AMBP: alpha-1-microglobulin/bikunin precursor, CD40L: CD40 ligand, DECR1: 2,4-dienoyl CoA reductase 1, FCGR2B: IgG Fc fragment low affinity receptor, GIF: gastric intrinsic factor, LEP: leptin, MARCO: macrophage receptor with collagenous structure, NPPB: atrial natriuretic factor, REN: renin, THPO: thrombopoietin, TNSF14: tumor necrosis factor superfamily member 14.

Protein Code According to String	Protein Code According to Olink	% of Samples in Group with No Complications	% of Samples in Group with Complications	Change of Protein Abundance	Biological Function	Role in Wound Healing
CD40LG	CD40-L	74	45	decrease	cytokine–cytokine receptor interaction, receptor binding *	lack of CD40L leads to excessive collagen deposition
ADAMTS13	ADAM-TS13	68	45	decrease	receptor binding *	lack of ADAMTS13 increases the extravasation of neutrophils
GIF	GIF	68	45	decrease	cyanocobalamin absorption #	not identified yet
FGF23	FGF-23	89	55	decrease	regulation of immune system process, regulation of MAPK cascade *	not FGF23 but other FGFs facilitate wound healing
CD84	CD84	74	27	decrease	modulation of the activation and differentiation of immune cells, regulation and interconnection of innate and adaptive immune response #	not identified yet
REN	REN	84	55	decrease	regulation of MAPK cascade, receptor binding *	depending on the conditions activates or inhibits wound healing
DECR1	DECR1	95	73	decrease	Metabolism #	not identified yet
AMBP	AMBP	84	64	decrease	regulation of immune system process, regulation of MAPK cascade *	AMBP deficiency is related to significantly more pathological-appearing cells in airway injury
FCGR2B	IgG Fc receptor II-b	47	18	decrease	regulation of immune system process *	not identified yet
THPO	THPO	42	0	decrease	regulation of immune system process, regulation of MAPK cascade, receptor binding *	THPO receptor agonist causes slight delay in wound healing
MARCO	MARCO	42	9	decrease	regulation of immune system process *	not identified yet
UGGT1	GT	26	0	decrease	protein folding quality control #	not identified yet
NPPB	BNP	47	18	decrease	receptor binding *	promotes wound healing
LEP	LEP	79	36	decrease	regulation of immune system process, regulation of MAPK cascade, cytokine–cytokine receptor interaction *	promotes wound healing
CA5A	CA5A	32	0	decrease	Metabolism #	lower levels of carbonic anhydrase were detected in keloid scars
TNFSF14	TNFSF14	100	73	decrease	regulation of immune system process, cytokine–cytokine receptor interaction, receptor binding *	lack of TNFSF14 helps the accumulation of immune cells, increases cytokine levels, impairs wound healing
CCL3	CCL3	89	64	decrease	regulation of immune system process, regulation of mitogen-activated protein kinase cascade, cytokine–cytokine receptor interaction, receptor binding *	helps macrophage recruitment; decreased CCL3 levels were observed in impaired wound healing
IL17C	IL-17C	37	64	increase	cytokine–cytokine receptor interaction #	overexpression is observed in psoriasis
IL20RA	IL-20RA	53	82	increase	cytokine–cytokine receptor interaction *	promotes corneal epithelial healing
IL10RA	IL-10RA	5	36	increase	cytokine–cytokine receptor interaction *	promotes survival of myeloid cells
TNFSF11	TRANCE	16	36	increase	cytokine–cytokine receptor interaction*, cell proliferation #	not identified yet
ARTN	ARTN	47	91	increase	neurotrophic factor, ligand for the RET receptor #	facilitates wound healing
FGF19	FGF-19	37	64	increase	regulation of bile acid synthesis #	not FGF19 but other FGFs facilitate wound healing

## Supplementary Materials

Supplementary materials can be found at http://www.mdpi.com/1422-0067/19/12/4096/s1.
