# Supplementary material for: Wound-Healing Markers Revealed by Proximity Extension Assay in Tears of Patients following Glaucoma Surgery
Source: Int J Mol Sci. 2018 Dec 18;19(12):4096. doi: 10.3390/ijms19124096 (PMC6321131; doi:10.3390/ijms19124096)
Supplement: Supplementary file 1 [file ijms-19-04096-s001.zip › table caption.rtf]

TableS1. Proteins belonging to the Olink panels used. The protein name, protein code, Uniprot ID and Olink ID along with the name of the panel is given in case of each analyzed protein.
TableS2. Raw data obtained by proximity extension assay. The normalized protein expression (NPX) values corresponding for the examined proteins for each individual sample are presented. For samples that did not pass quality control, data are highlighted in red. Values below limit of detection (LOD) are highlighted with red background and the LOD value is given. In case of the examined proteins the protein code, the Uniprot ID and the Olink ID is provided along with data corresponding to the panel used. In case of each protein the LOD, missing data frequency and the detectability is indicated as well.
TableS3. Patient data. 
TableS4. Result of qualitative and quantitative analyses. The number and percentage of cases where the protein was detected in case of each analyte in the groups with our without complication is shown along with the mean NPX values and the results of Mann-Whitney U test. p<0.05 was considered as statistically significant.
